# The Impact of Different Animal-Derived Protein Sources on Adiposity and Glucose Homeostasis during *Ad Libitum* Feeding and Energy Restriction in Already Obese Mice

**DOI:** 10.3390/nu11051153

**Published:** 2019-05-23

**Authors:** Lene Secher Myrmel, Kristin Røen Fauske, Even Fjære, Annette Bernhard, Ulrike Liisberg, Astrid Elise Hasselberg, Jannike Øyen, Karsten Kristiansen, Lise Madsen

**Affiliations:** 1 Institute of Marine Research, NO-5817 Bergen, Norway; kristin.roen.fauske@helse-bergen.no (K.R.F.); even.fjaere@hi.no (E.F.); annette.bernhard@hi.no (A.B.); ulrike.liisberg@hvl.no (U.L.); astrid.hasselberg@hi.no (A.E.H.); jannike.oyen@hi.no (J.Ø.); lise.madsen@hi.no (L.M.); 2 Department of Biomedicine, University of Bergen, NO-5020 Bergen, Norway; 3 Laboratory of Genomics and Molecular Biomedicine, Department of Biology, University of Copenhagen, DK-2100 Copenhagen, Denmark; kk@bio.ku.dk

**Keywords:** obesity, diet, dietary protein source, macronutrient composition, weight loss, glucose tolerance, insulin sensitivity, mice

## Abstract

Low-fat diets and energy restriction are recommended to prevent obesity and to induce weight loss, but high-protein diets are popular alternatives. However, the importance of the protein source in obesity prevention and weight loss is unclear. The aim of this study was to investigate the ability of different animal protein sources to prevent or reverse obesity by using lean or obese C57BL/6J mice fed high-fat/high-protein or low-fat diets with casein, cod or pork as protein sources. Only the high-fat/high-protein casein-based diet completely prevented obesity development when fed to lean mice. In obese mice, *ad libitum* intake of a casein-based high-fat/high-protein diet modestly reduced body mass, whereas a pork-based high-fat/high-protein diet aggravated the obese state and reduced lean body mass. Caloric restriction of obese mice fed high-fat/high-protein diets reduced body weight and fat mass and improved glucose tolerance and insulin sensitivity, irrespective of the protein source. Finally, in obese mice, *ad libitum* intake of a low-fat diet stabilized body weight, reduced fat mass and increased lean body mass, with the highest loss of fat mass found in mice fed the casein-based diet. Combined with caloric restriction, the casein-based low-fat diet resulted in the highest loss of fat mass. Overall, the dietary protein source has greater impact in obesity prevention than obesity reversal.

## 1. Introduction

Weight loss is the main target in order to reduce the risk for diabetes in humans [1]. Conventional weight loss strategies typically recommend overweight and obese persons to reduce fat intake in order to reduce the energy intake by 500–750 calories [2]. However, high-protein (HP) diets are becoming a popular alternative to energy restriction for body weight maintenance and weight loss in humans. In addition to an increased satiating effect, HP diets are reported to increase energy expenditure [3,4,5]. However, the long term health effects of HP diets, in particular when combined with high-fat (HF) intake, remain controversial [6], and unless the dietary intervention is combined with energy restriction, the long term efficiency of HP diets to induce weight loss in humans is inconclusive, as demonstrated in systematic reviews and meta-analyses [7,8,9].

Whereas human trials mainly have examined the ability of HP diets to induce weight loss in obese subjects, the effects of HP diets in rodents have primarily been investigated in relation to the prevention of obesity development. A number of animal trials have reported that HF/HP diets efficiently attenuate and even prevent the development of obesity [10,11,12,13,14,15,16,17,18,19], but the ability of HF/HP diets to reverse obesity in rodents is far less clear. An important issue regarding HF/HP diets relates to reports indicating that the dietary fat content, rather than the obese state, may be a key determinant for dysregulated glucose homeostasis [20,21,22]. Whereas it is well documented that weight loss is induced by caloric restriction [23], low-fat (LF) diets [24] and exercise [25] also lead to improved glucose homeostasis and insulin sensitivity in mice. Still, it is not yet known if HF/HP diets ameliorate or worsen dysregulated glucose metabolism in already obese mice.

In the majority of the animal studies demonstrating that a HP content can prevent obesity, casein or whey is used as the sole protein source [12,13,14,15,16,17,18,19]. However, both casein and whey appear to have anti-obesogenic properties and may not be representative. We have previously observed that mice fed HF/HP diets based on casein remain lean, but if the protein source is chicken, pork or beef, weight gain is greatly increased, reflecting an increase in fat mass [26]. An association between maintenance of classical brown morphology in the interscapular brown adipose tissue and obesity prevention has been observed, but a direct causal link between these findings has not yet been demonstrated. Furthermore, it is not known whether casein is able to reverse the white phenotype of the interscapular fat depot in obese mice.

Although a number of human trials have examined the ability of HP diets to induce weight loss in obese subjects [7,8,9], the importance of the protein source remains unclear. In several of these trials, the dietary intervention was combined with energy restriction, and differences in design made it difficult to arrive at solid conclusions [3]. Nonetheless, when consumed at regular levels in habitual diets, different protein sources appear to have different obesogenic properties in humans. Epidemiological and prospective studies suggest that while a high intake of meat from terrestrial sources, in particular red meat, is associated with weight gain, intake of marine, dairy and vegetarian protein sources is associated with protection against becoming overweight [27,28]. Hence, it remains an open question whether protein sources are of more importance in preventing weight gain than in diets used to achieve weight loss, in particular if energy is restricted.

The present global obesity epidemic emphasizes the need for effective weight loss strategies in addition to effective methods for weight gain prevention. Given the popularity of HP diets, we here aimed to investigate if HP diets that can attenuate obesity development are able to reverse obesity in mice, or if concomitant energy restriction is a prerequisite for weight loss. Furthermore, we aimed to systematically investigate the impact of different animal protein sources on weight loss, glucose tolerance and insulin sensitivity when combined with different macronutrient compositions and energy access. The use of mice as a model allows for a systematic investigation of the importance of the protein source in putative weight loss induced by dietary interventions and the evaluation of the possible detrimental or beneficial effects of the diets on glucose tolerance and insulin sensitivity. We demonstrate that the dietary protein source is of higher importance in the development of obesity when fed to lean mice than when fed to already obese mice *ad libitum*. Unless accompanied with energy restriction, HF/HP diets generally do not reverse obesity and dysregulated glucose homeostasis efficiently. Finally, we demonstrate that the protein source is of less importance when the dietary interventions are combined with energy restriction, and the regulation of glucose homeostasis appears to follow the degree of obesity rather than the dietary fat content.

## 2. Materials and Methods

### 2.1. Ethical Statement

All animal experiments were approved by the Norwegian Animal Research Authority (FOTS id.nr 5358). Animal handling and experimental protocols were performed in accordance with the guidelines of the national authorities and the European Convention for the Protection of Animals used for scientific purposes (regulation on the use of animals in research, Ministry of Agriculture and Food, 1 July 2015; according to Directive 2010/63/EU of the European Parliament and of the Council of 22 September 2010). No adverse effects were observed during the experiments.

### 2.2. Animal Experiments

In all animal experiments, C57BL/6J male mice, 8 weeks old, obtained from Taconic Europe (Ejby, Denmark) were used. Body weight was recorded every week and fresh water was provided twice a week. Body composition analyses were, in addition, measured at the start and at the designated time-points for each animal experiment, as described in Section 2.4. All mice fed *ad libitum* were given new feed three times each week, and feed leftovers were recorded. The mice fed with 30% energy restriction were fed each day in that period to avoid prolonged fasting periods between feeding, and feed leftovers were recorded. All animals were housed in a thermoneutral environment (29 ± 1 °C) with 50% relative humidity and a 12-h light/dark cycle. The mice were sacrificed during 4% isoflurane anesthesia (Isoba vet, Schering-Plough A/S, Farum, Denmark) by cardiac puncture and adipose tissues were dissected out, snap-frozen in liquid nitrogen and stored at −80 °C.

#### 2.2.1. First Animal Experiment

The first animal experiment was performed using 45 lean male mice, 8 weeks old. After one week of acclimatization, body mass composition was determined and mice were divided into groups with similar body weight (25 ± 0.7 g), fat mass (2.5 ± 0.2 g) and lean mass (18.5 ± 0.6 g). The mice were fed the experimental diets (Table 1) *ad libitum*, including a LF diet, a high-fat/high-sucrose (HF/HS) diet and HF/HP diets based on either casein, cod or pork for 12 weeks (*n* = 9). All animals were subjected to an oral glucose tolerance test (OGTT) and an insulin tolerance test (ITT) after 10 and 11 weeks, respectively, as further described in Section 2.5. In week 6, total feces was collected during 7 days from all the individual cages, weighed and analyzed for total nitrogen and total fat content as previously described [29]. Apparent digestibility of nitrogen and fat were determined using the total fat and nitrogen content in the collected feces, in addition to the total dietary fat and nitrogen intake during the 7 days of feces collection.

#### 2.2.2. Second Animal Experiment

The second animal experiment was performed using 40 already obese male mice. In order to achieve obesity (mean body weight: 41.1 ± 0.5 g, mean fat mass: 17.0 ± 0.3 g), 8 week old mice were fed a HF/HS diet *ad libitum* (Table 1) for 8 weeks. Body composition was determined in all the obese mice and used to divide the mice into groups with similar body weight, fat mass and lean mass. The obese mice received the different experimental diets *ad libitum*, including HF/HP diets based on casein, cod or pork, or continued receiving a HF/HS diet for 6 weeks. After 4 and 5 weeks on the experimental diets, all animals were subjected to an ITT and an OGTT as further described in Section 2.5.

#### 2.2.3. Third Animal Experiment

The third animal experiment was performed using 52 already obese male mice. In order to achieve obesity (mean body weight: 43.1 ± 0.5 g, mean fat mass: 17.0 ± 0.2 g), 8 week old mice were fed a HF/HS (Table 1) *ad libitum* for 9 weeks. Body composition was determined and used to divide the obese mice into groups with similar body weight, fat mass and lean mass. The obese mice received the different experimental diets with a 30% energy restriction, including HF/HP diets based on casein, cod or pork, or continued on a HF/HS diet *ad libitum* for 6 weeks. Energy restricted mice received 1.75 g of feed every day. After 4 and 5 weeks on the experimental diets, all animals were subjected to an ITT and an OGTT as further described in Section 2.5.

#### 2.2.4. Fourth Animal Experiment

The fourth animal experiment was performed using 40 already obese male mice. In order to achieve obesity (mean body weight: 41.8 ± 0.7 g, mean fat mass: 17.9 ± 0.6 g), 8 week old mice were first fed a very high-fat (VHF) diet *ad libitum* (Ssniff EF R/M acc D12492, Soest, Germany) for 18 weeks. Body composition was determined and used to divide the obese mice into groups with similar body weight, fat mass and lean mass. The obese mice received the different experimental diets *ad libitum*, including LF diets based on casein, cod or pork (Table 1), or continued on a VHF diet *ad libitum* for 6 weeks. After 4 and 5 weeks on the experimental diets, all animals were subjected to an ITT and an OGTT as further described in Section 2.5.

#### 2.2.5. Fifth Animal Experiment

The fifth animal experiment was performed using 52 already obese male mice. In order to achieve obesity (mean body weight: 42.0 ± 0.5 g, mean fat mass: 18.0 ± 0.5 g), 8 week old mice were first fed a VHF diet *ad libitum* (Ssniff EF R/M acc D12492, Soest, Germany) for 13 weeks. Body composition was determined and used to divide the obese mice into groups with similar body weight, fat mass and lean mass. The obese mice received the different experimental diets with a 30% energy restriction, including LF diets based on casein, cod or pork (Table 1), or continued on a VHF diet *ad libitum* for 6 weeks. Energy restricted mice received 2.0 g feed every day. After 4 and 5 weeks on the experimental diets, all animals were subjected to an ITT and an OGTT as further described in Section 2.5.

### 2.3. Animal Diets

All HF/HP and LF diets based on different protein sources (Table 1) were prepared by the use of either casein powder (C8654 SIGMA, Merck, Darmstadt, Germany), fillets from frozen cod (Lerøy Alfheim AS, Bergen, Norway) or fresh pork sirloins (H. Bragstad A/S, Bergen, Norway). The cod fillets and pork sirloins were heat treated (70 °C), freeze dried and powdered before being added to the diets in amounts equal to 200 g crude protein/kg for the LF diets and 400 g crude protein/kg for the HF/HP diets (Table 1), calculated from measurements of nitrogen content in the powders as described previously [30]. In addition, the endogenous total fat content in the protein powders was measured as described previously [29] and the diets were adjusted with corn oil to achieve an equal amount of total fat in the diets.

### 2.4. Body Composition Measurements

Whole-body composition, fat mass, lean mass and free water were measured by noninvasive scanning that uses a time-domain nuclear magnetic resonance system (Bruker Minispec LF50 Body Composition Analyzer mq 7.5 (Bruker Optic GmbH, Ettlingen, Germany)) as previously described [31].

### 2.5. Glucose- and Insulin Tolerance Tests

An OGTT and ITT were performed in all the animals at designated time-points during the feeding trials. The OGTT was performed in fasted (6 h) animals which received 3 mg glucose per g lean body mass by oral gavage. The ITT was performed in fed animals receiving an intraperitoneal injection of 1 U insulin per kg lean body mass. In both tests, glucose levels were measured in blood collected from the tail vein at the indicated time points using a glucometer (Ascensia Contour, Bayer, Norway).

### 2.6. Histology and Immunohistochemistry

Parts of the adipose tissue, inguinal white adipose tissue (iWAT) and interscapular brown adipose tissue (iBAT), were excised and fixated overnight in 4% formaldehyde dissolved in 0.1 M phosphate buffer at 4 °C. After fixation, the tissues were dehydrated, cleared in xylene and embedded in paraffin. Sections (5 µm) of the paraffin embedded tissues were stained with hematoxylin and eosin (H&E) for further morphological evaluations using a Nikon Eclipse Ti microscope (Nikon Instruments Inc., New York, USA) and micrographs captured by a Nikon DS-Fi3 camera (Nikon Instruments Inc., New York, USA). Immunohistochemical detection of uncoupling protein 1 (UCP1) in iBAT was performed as described previously [20] and quantified using Image J, Fiji [32].

### 2.7. Quantitative Reverse Transcriptase PCR (qRT-PCR)

Total RNA was purified from iWAT and iBAT and used to further synthesize complementary DNA by reverse transcription PCR, and real time PCR was performed, as previously described [33]. Primers specific for the designated genes were designed using Primer Express 2.0 (Applied Biosystems) and the sequences are listed in Table S1. The relative expression levels of target genes were normalized to the gene expression of TATA box binding protein (*Tbp*).

### 2.8. Statistical Analysis

The data presented in graphs are mean ± standard error of the mean (SEM). Homogeneity of variances was tested using the Brown–Forsythe test and the normality of distribution was tested using the D’Agostine–Pearson omnibus normality test. No significant differences were detected. An ANOVA with an uncorrected Fisher’s Least Significant Difference (LSD) for multiple comparisons was performed to compare the experimental groups. Different letters denote statistical differences (*P* < 0.05). All statistical tests and calculations of area under and over curves were performed using GraphPad Prism 7 (GraphPad Software, Inc, San Diego, USA).

## 3. Results

### 3.1. The Protein Source Determines the Impact of High Protein Diets on the Development of Obesity, Glucose Tolerance and Insulin Sensitivity in Lean Mice

**Figure 1:**
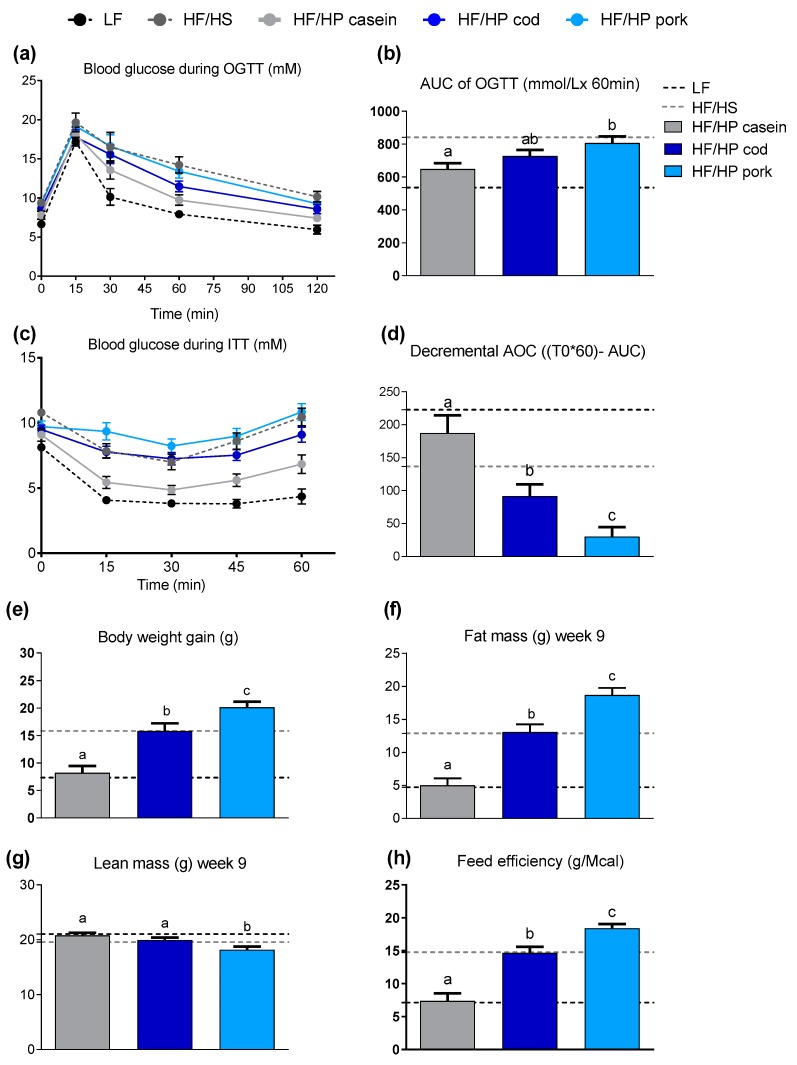
(**a**) Blood glucose levels during the oral glucose tolerance test (OGTT) performed in week 10 of feeding *ad libitum* high-fat/high-protein (HF/HP) diets based on different protein sources to lean mice, in addition to one group fed a low-fat (LF) diet and one fed a high-fat/high-sucrose (HF/HS) diet. (**b**) Area under the curve (AUC) for the OGTT. (**c**) Glucose levels during the insulin tolerance test (ITT) in week 11 and (**d**) the decremental area over the curve (AOC). (**e**) Body weight gain after 10 weeks, (**f**) fat mass and (**g**) lean mass in week 9 and (**h**) feed efficiency after 10 weeks. Data are presented as mean ± standard error of the mean (SEM) (*n* = 8–9) and different letters denote significant differences (*P* < 0.05) by one-way ANOVA using uncorrected Fisher’s Least Significant Difference (LSD) multiple comparison.

First, we aimed to evaluate glucose tolerance and insulin sensitivity, and to verify the different obesogenic potential of casein, cod and pork as protein sources in HF/HP diets. Hence, we fed lean male mice HF/HP diets based on proteins from either casein, cod or pork (Table 1) for 12 weeks. Compared with casein-fed mice, pork-fed mice had a lower lean mass, larger adipocyte size, reduced glucose tolerance and insulin sensitivity (Figure 1a–d and Figure S1a,b), while cod-fed mice had a higher lean mass and increased insulin sensitivity compared with pork-fed mice (Figure 1c,d,g). As previously demonstrated [26], the HF/HP diet with pork was highly obesogenic (Figure 1e,f). Mice fed the HF/HP diet containing casein remained as lean as control mice fed a LF reference diet, whereas body weight gain and fat mass of mice fed the cod-containing diet were higher compared with casein and lower in comparison with chicken. The feed intake was not different (Figure S2a), and feed efficiency reflected the weight gain (Figure 1h). As fat digestibility was higher only in mice fed casein, and nitrogen digestibility was not affected by the protein source (Figure S2b,c); these parameters cannot explain the differences in obesity development between the protein sources.

In agreement with earlier studies [26], the obese phenotype of the pork-fed mice was accompanied with a profound “whitening” of the classical iBAT (Figure S1d), whereas mice fed casein maintained a classic brown adipocyte morphology with multilocular lipid droplets and increased protein expression of UCP1 (Figure S1e). Hence, the ability of casein-based HF/HP diets to enable the maintenance of a brown phenotype in iBAT and prevent obesity was verified. Furthermore, mice fed the casein-based diet had a higher expression of *Ucp1*, deiodinase iodothyronine type II (*Dio2*) and peroxisome proliferator-activated receptor-g coactivator 1a (*Ppargc1a*) in iWAT compared with mice fed pork (Figure S1c), suggesting an increased browning of the white adipose tissue. In cod-fed mice, expression levels of brown adipose tissue (BAT) marker genes in iWAT, as well as UCP1 protein expression levels in iBAT were intermediate compared with the levels of casein- and pork-fed mice, but not significantly different from either. Thus, the profound difference in adiposity between mice fed HF/HP diets with casein or pork as protein sources was accompanied by differences in iBAT morphology, glucose tolerance and insulin sensitivity.

### 3.2. The Impact of Protein Source in Obese Mice Fed High Protein Diets

#### 3.2.1. High Protein Diets Fed *Ad Libitum* to Obese Mice

**Figure 2:**
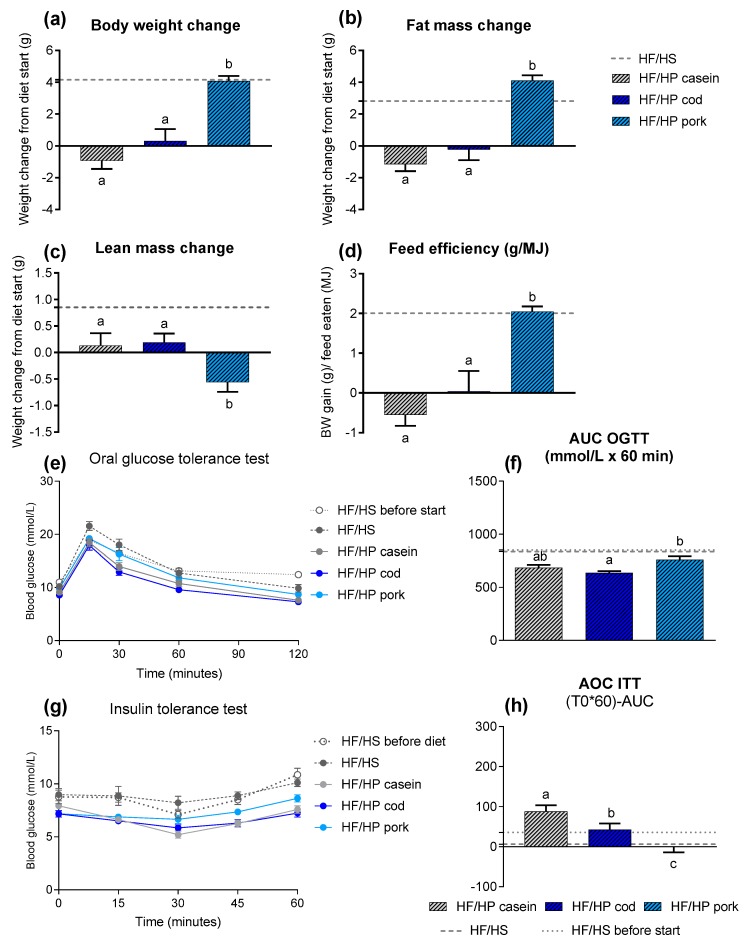
(**a**) Body weight change, (**b**) fat mass change and (**c**) lean mass change after 6 weeks of feeding high-fat/high-protein (HF/HP) diets based on different protein sources *ad libitum* to already obese mice, in addition to one group fed the obesogenic high-fat/high-sucrose (HF/HS) diet. (**d**) Feed efficiency (g/MJ) from 6 weeks of HF/HP feeding. (**e**) Blood glucose levels during the oral glucose tolerance test (OGTT) performed in week 4 and (**f**) area under the curve (AUC) for the OGTT. (**g**) Blood glucose levels during the insulin tolerance test (ITT) in week 5 and (**h**) area over the curve (AOC) for the ITT. Data are presented as mean ± SEM (*n* = 9–12) and different letters denote significant differences (*P* < 0.05) by one-way ANOVA using uncorrected Fisher’s LSD multiple comparison.

In order to investigate the ability of HP diets to reverse obesity, obese mice (body weight: 41.1 ± 0.5 g, fat mass: 17.0 ± 0.3 g, lean mass: 18.1 ± 0.2 g) were fed HF/HP diets based on proteins from either casein, cod or pork (Table 1) for 6 weeks *ad libitum*. Although a casein-based HF/HP diet is able to prevent the development of obesity, feeding obese mice this diet led to only a modest decrease in body weight (0.93 g) and fat mass (1.15 g). Whilst an *ad libitum* intake of a HF/HP diet based on pork further increased body and fat mass in the obese mice (Figure 2a,b), *ad libitum* intake of a HF/HP diet based on cod prevented further weight and fat mass gain. Thus, although feeding obese mice HF/HP diets for 6 weeks did not reverse obesity, a differential impact of the protein source on adiposity was still evident. Histological examination and immunostaining of UCP1 in iBAT revealed a white phenotype in samples from all groups, including samples collected from obese mice prior to the dietary intervention (Figure S3a,b). In line with this, no differences in the expression of *Ucp1*, *Dio2* and *Ppargc1a* in iBAT were evident (Figure S4a). Hence, although a casein-based HF/HP diet appears to enable maintenance of a brown phenotype in iBAT, this diet cannot reverse whitening of the tissue. However, the expression of *leptin*, a gene normally expressed at high levels in white adipocytes, was significantly higher in the brown adipose tissue of pork-fed mice. The adipocytes from the iWAT tended to be enlarged in mice fed HF/HP diets based on pork (*P* = 0.06) compared with cod (Figure S3c,d). In line with this, expression of genes related to browning (*Ucp1, Dio2, Ppargc1a*) was low in mice fed the HF/HP diet based on pork (Figure S4b). Increased expression of these marker genes in iWAT was evident for all the mice with a high casein intake, both after feeding lean and obese mice *ad libitum*, and in comparison with pork and/or cod (Figures S1c and S4b).

A reduction in lean mass was observed after 6 weeks of intervention in mice fed the pork-based diet (Figure 2c). The reduction in lean mass was also significantly higher in mice fed pork, compared with mice fed either casein or cod, which preserved the lean mass throughout the intervention. Feed efficiency mirrored the change in body weight and fat mass, demonstrating a greater feed efficiency in mice fed pork compared with mice fed either casein or cod (Figure 2d).

Following the change in body weight, pork-fed mice had the highest area under the curve for the OGTT and were less sensitive to insulin compared with casein- or cod-fed mice (Figure 2e–h). In comparison to their state prior to the intervention, all mice fed the HF/HP diets demonstrated an improvement in glucose tolerance according to the area under the curve for the OGTT, whereas only mice fed HF/HP diets based on casein had an improved insulin sensitivity according to the area over the curve for the ITT.

#### 3.2.2. High Protein Diets Combined with Calorie Restriction

**Figure 3:**
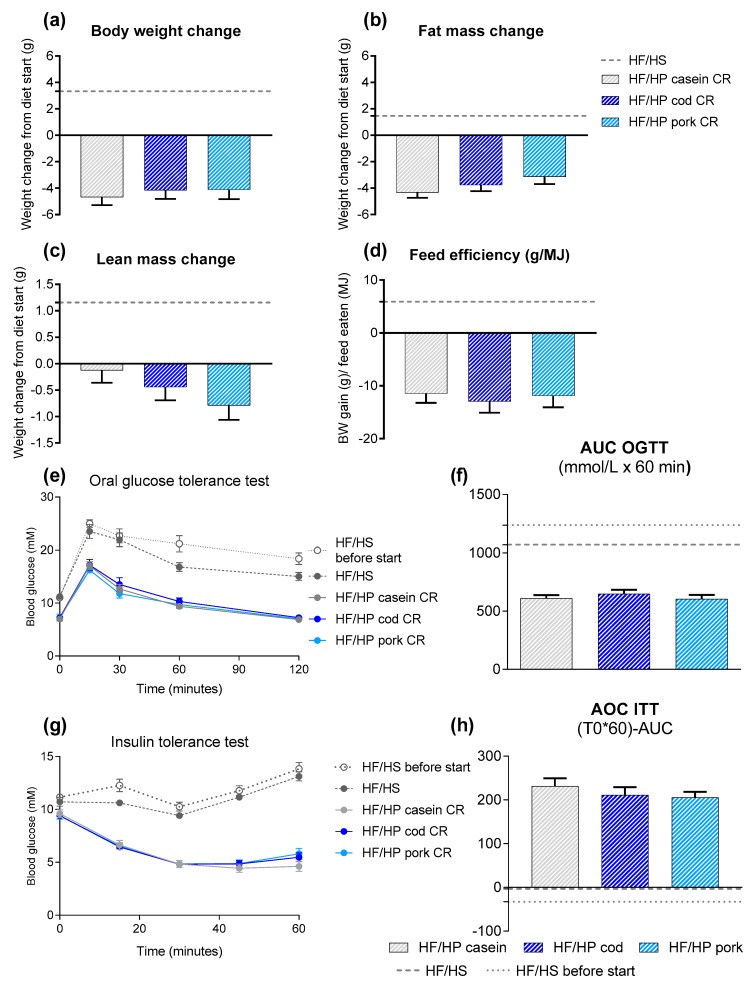
(**a**) Body weight change, (**b**) fat mass change and (**c**) lean mass change after 6 weeks of feeding high-fat/high-protein (HF/HP) diets based on different protein sources with 30 per cent calorie restriction (CR) to already obese mice, in addition to one group fed the obesogenic high-fat/high-sucrose (HF/HS) diet *ad libitum*. (**d**) Feed efficiency (g/MJ) from 6 weeks of HF/HP feeding. (**e**) Blood glucose levels during the oral glucose tolerance test (OGTT) performed in week 4 and (**f**) area under the curve (AUC) for the OGTT. (**g**) Blood glucose levels during the insulin tolerance test (ITT) in week 5 and (**h**) area over the curve (AOC) for the ITT. Data are presented as mean ± SEM (*n* = 9–13) and different letters denote significant differences (*P* < 0.05) by one-way ANOVA using uncorrected Fisher’s LSD multiple comparison.

Next, we investigated if the protein sources influenced the ability of HF/HP diets to reverse obesity during caloric restriction. For this purpose, obese mice (body weight: 43.1 ± 0.5 g, fat mass: 17.0 ± 0.2 g, lean mass: 19.8 ± 0.2 g) were fed the same HF/HP diets combined with 30% calorie restriction for 6 weeks. Body mass, fat mass, lean mass and feed efficiency were reduced in all mice fed HF/HP diets with calorie restriction with no further differences being evident between the different protein sources (Figure 3a–d). Morphological examination of iBAT in mice fed either of the diets revealed a brown phenotype, whereas the expression levels of classical brown adipocyte marker genes were not influenced by the dietary protein source in iBAT (Figures S4a and S5b). The expression levels of genes related to browning (*Ucp1, Dio2, Ppargc1a*) were not higher in iWAT from casein-fed mice (Figure S4b). In fact, during caloric restriction, expression of these genes was slightly elevated in cod-fed mice when compared with mice fed casein.

Consistent with body mass and fat mass loss, all the HF/HP diets induced an improvement in glucose tolerance and insulin sensitivity in the obese mice compared with before the diet started and to the mice that continued receiving the obesogenic reference diet (Figure 3e–h). However, there were no differences in glucose tolerance or insulin sensitivity between mice fed the different protein sources with calorie restriction. Thus, the protein source of the high-fat/high-protein (HF/HP) diets appeared to be of importance in prevention of weight gain when given *ad libitum* to lean and obese mice, but this was absent in combination with caloric restriction.

### 3.3. The Impact of Protein Source on Weight Loss in Obese Mice Given Low Fat Diets

**Figure 4:**
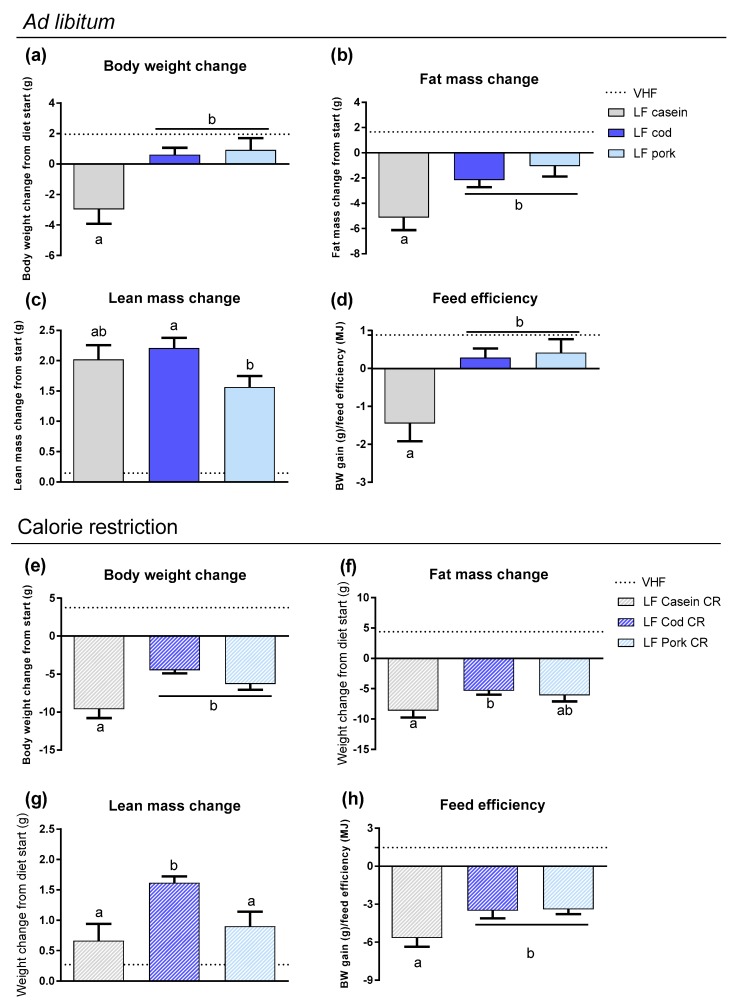
(**a**) Body weight change, (**b**) fat mass change and (**c**) lean mass change after 6 weeks of feeding low-fat (LF) diets based on different protein sources *ad libitum* to already obese mice, in addition to one group fed the obesogenic very high-fat (VHF) diet *ad libitum*. (**d**) Feed efficiency (g/MJ) from 6 weeks of LF feeding. (**e**) Body weight change, (**f**) fat mass change and (**g**) lean mass change after 6 weeks of feeding LF diets based on different protein sources with 30 per cent calorie restriction (CR) to already obese mice, in addition to one group fed the obesogenic VHF diet *ad libitum*. (**h**) Feed efficiency (g/MJ) from 6 weeks of LF feeding. Data are presented as mean ± SEM (*n* = 9–13) and different letters denote significant differences (*P* < 0.05) by one-way ANOVA using uncorrected Fisher’s LSD multiple comparison.

To evaluate the importance of the protein source during *ad libitum* feeding vs. caloric restriction in weight loss diets with regular protein amount, LF diets based on casein, cod or pork were prepared. First, obese mice (body weight: 41.8 ± 0.7 g, fat mass: 17.9 ± 0.6 g, lean mass: 18.8 ± 0.1 g), were fed the LF diets *ad libitum* for 6 weeks. As a reference, one group of mice was continuously fed the obesogenic VHF diet. The body mass of all mice fed LF diets (Table 1) *ad libitum* was stabilized or modestly decreased throughout the 6 week intervention period, with a reduction in body weight of mice fed casein compared with cod or pork (Figure 4a). This pattern was also reflected in their feed efficiency (Figure 4d). Despite a stable body weight, fat mass was reduced in all LF-fed mice, with the highest fat mass loss found in mice fed casein (Figure 4b). An increase in lean mass was evident in all mice fed LF diets, with a higher increase in the mice fed LF diets based on cod compared with pork (Figure 4c). Hence, the protein source was a decisive factor for changes in body composition of mice fed LF diets *ad libitum*.

Next, we investigated whether differences between the protein sources in LF diets were revoked in combination with caloric restriction. Hence, a new set of obese mice (body weight: 42.0 ± 0.5 g, fat mass: 18.0 ± 0.5 g, lean mass: 18.9 ± 0.1 g) were fed LF diets with casein, cod or pork combined with 30% calorie restriction for 6 weeks. As expected, body mass and fat mass loss were observed in all groups (Figure 4e,f). In contrast to the combination of HF/HP diets with energy restriction, mice fed a casein-based LF diet with calorie restriction demonstrated a higher body weight loss compared with mice fed cod or pork. This pattern was also reflected in their feed efficiency (Figure 4h). Reduction in body mass was mainly caused by fat mass loss, with higher fat mass loss in mice fed casein compared with cod. Overall, an increase in lean mass was evident with the LF intake, despite calorie restriction, with a higher lean mass gain in mice fed cod compared with casein or pork (Figure 4g).

No distinct expression pattern was observed for the marker genes related to browning measured in iWAT of LF fed mice *ad libitum* or with calorie restriction (Figure S6a). Mice fed a casein-based LF diet demonstrated an upregulated *Ucp1* expression in iBAT compared with mice fed cod or pork, independent of feed access. In addition, the expression of glycerol kinase (*Gk*) was upregulated in iBAT of casein-fed mice compared with mice fed cod and pork diets *ad libitum* or with calorie restriction (Figure S6b), which has been demonstrated to play an important role for the thermogenic function/capacity of brown adipocytes [34].

**Figure 5:**
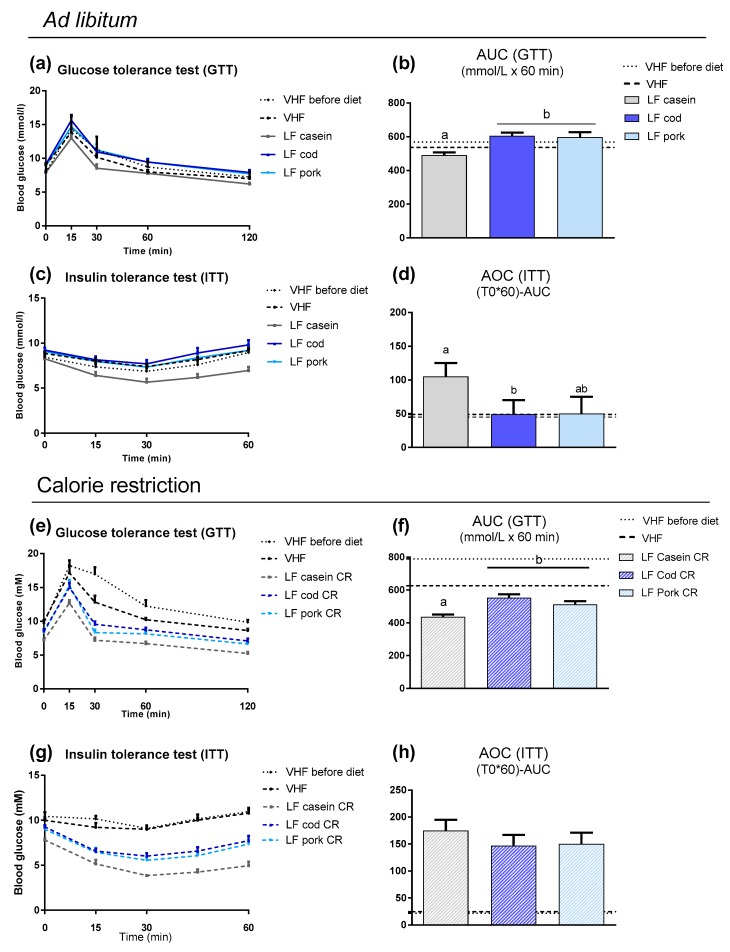
(**a**) Blood glucose levels during the oral glucose tolerance test (OGTT) performed in week 4 of feeding low-fat (LF) diets based on different protein sources *ad libitum* to already obese mice, in addition to one group fed the obesogenic very high-fat (VHF) diet *ad libitum*. (**b**) Area under the curve (AUC) for the OGTT. (**c**) Glucose levels during the insulin tolerance test (ITT) in week 5 and (**d**) the decremental area over the curve (AOC). (**e**) Blood glucose levels during the OGTT performed in week 4 feeding LF diets based on different protein sources with 30 percent calorie restriction (CR) to already obese mice, in addition to one group fed the obesogenic VHF diet *ad libitum*. (**f**) AUC for the OGTT. (**g**) Glucose levels during the ITT in week 5 and (**h**) the decremental AOC. Data are presented as mean ± SEM (*n* = 9–13) and different letters denote significant differences (*P* < 0.05) by one-way ANOVA using uncorrected Fisher’s LSD multiple comparison.

Despite fat mass loss and lean mass increase in all LF-fed *ad libitum* groups, glucose tolerance was not improved and insulin sensitivity was improved only in casein-fed mice (Figure 5a–d). Consistent with the differences in fat mass, all energy restricted LF-fed mice had improved glucose tolerance and insulin sensitivity (Figure S5e–h). In both *ad libitum* and energy restricted groups, the glucose tolerance tests demonstrated that mice fed LF diets based on casein had improved glucose tolerance compared with mice fed cod or pork. This was also in accordance with greater insulin-stimulated glucose responses in mice fed a LF diet *ad libitum* with casein compared with cod, although not compared with pork. Hence, the protein source was also of importance for glucose metabolism during LF intake. However, the impact of the protein source on glucose metabolism was more pronounced when mice were fed *ad libitum* than under energy restriction.

## 4. Discussion

Using systematically designed mouse trials, we here demonstrated that the different dietary animal protein sources are of more importance in preventing weight gain than in diets used to achieve weight loss, particularly if energy is restricted. In line with earlier observations [26], different protein sources demonstrated profound differences in their ability to modulate obesity development in lean mice fed HF/HP diets. Whereas mice fed the casein-based diet remained lean, mice fed pork developed obesity and mice fed cod were in-between. Similarly, in experiments using obesogenic diets with regular protein amounts, it was demonstrated that protein from vegetable sources, milk protein and proteins from seafood are less obesogenic than proteins from terrestrial animals [29,35,36,37]. These results are in line with human prospective studies indicating that an increased intake of protein from vegetarian sources, dairy and seafood protect against obesity, whereas a high intake of meat, in particular red meat, is associated with higher weight gain [27,28,38]. Of note, changes in most protein foods were inversely correlated with changes in carbohydrate at the baseline, and replacing protein-rich food for carbohydrate-rich foods was essential for long-term weight maintenance [28].

An important finding in this study is that, unless combined with energy restriction, HF/HP diets do not reverse obesity. When fed *ad libitum*, the obese state is modestly reduced when casein is used as the protein source and aggravated when pork is used. Meta-analyses of HP diets on weight loss in humans report modest or no effect [7,8,9]. In these human trials, different protein sources were used, and several interventions combined HP diets with energy restriction. Hence, it is tempting to speculate if the lack of consistency in results from human trials may relate to different designs of the human trials with respect to energy restriction and/or the possible impact of different protein sources when the diets are given *ad libitum*.

Casein is commonly used as the protein source in commercially available rodent feed. We here confirm the earlier observation [26] that casein is not a representative protein source. When given *ad libitum*, only the casein-based HF/HP diet was able to reduce body mass and fat mass in obese mice, whereas the pork-based diet aggravated the obese state. Furthermore, feeding obese mice a casein-based LF diet reduced the fat mass significantly more than cod- and pork-based LF diets. A recent evaluation from meta-analyses of observational studies and randomized controlled trials concluded that the intake of milk and dairy products can also improve body composition and facilitate weight loss in humans [39]. The anti-obesogenic effect of casein and whey may relate to their relatively high content of branched-chain amino acids (BCAAs). Inclusion of BCAAs in HF diets is known to attenuate obesity in mice (leucine) [10] and rats [40], and chronic elevated levels of BCAAs in mice are associated with increased energy expenditure [41].

We observed large differences in energy efficiency with an overall lower feed efficiency demonstrated in mice fed diets based on casein. Energy may be dissipated to the environment in the form of heat via UCP1, which uncouples oxidative phosphorylation in the inner mitochondrial membrane present in brown and brown-like adipocytes [42], and increased expression and activity of UCP1 protects against diet-induced obesity [43]. In line with the ability of casein intake to attenuate obesity development [26], the classic brown adipose tissue morphology and high UCP1 expression in iBAT were maintained after feeding lean mice a casein-based HF/HP diet. In already obese mice, a “whitening” of the iBAT was evident. When combined with energy restriction, all mice receiving HF/HP diets lost body and fat mass and the brown phenotype of iBAT was retrieved. However, the white phenotype of iBAT was not reversed by feeding obese mice a casein-based HF/HP diet *ad libitum*, and in line with this, only a modest reduction in body weight and fat mass was observed. This indicates a greater potential for casein-derived HF/HP diets to prevent obesity and “whitening” of the iBAT, than to reverse obesity and “whitening” of iBAT.

Compared with a pork-based HF/HP diet, the casein-based diet led to an increased expression of brown adipocyte marker genes in white adipose tissue (WAT). Both obese and lean mice fed the pork-based HF/HP diet had larger adipocytes in iWAT. Browning of WAT has been associated with attenuated diet-induced obesity in several genetic models [43], and prevention of browning is demonstrated to impair fat loss induced by calorie restriction in mice [44]. However, it has been argued that the quantitative contribution of the relatively low number of mitochondria in iWAT compared with iBAT is minor [45]. Although further studies are required to determine the potential importance of UCP1 in iBAT and/or iWAT, our results indicate that inclusion of casein in diets may prevent whitening of iBAT and induce browning of iWAT.

Combined with calorie restriction, all HF/HP diets induced weight and fat mass loss independently of the protein source. Similarly, we observed improved glucose tolerance and insulin sensitivity in energy restricted mice, independently of protein source in HF/HP-fed mice. Hence, our study is not in agreement with the assumption that the dietary fat content, rather than the obese state, may be a key determinant for dysregulated glucose homeostasis [20,21,22]. Our results are in agreement with a large human trial, demonstrating that a reduction in calorie intake induces weight loss independent of the dietary macronutrient composition in obese adults [46]. When combined with caloric restriction, all diets, including those with relatively HF content, improved lipid-related risk factors and fasting insulin levels [46]. In our study, a LF diet in combination with calorie restriction was more efficient for body weight and fat mass loss in mice when based on casein compared with cod and pork. This is in agreement with a study in obese adults demonstrating that dairy products increased body weight and fat mass loss induced by a standard energy restricted diet [47]. Hall et al. [48] demonstrated dietary fat restriction to induce a higher body fat loss in obese adults compared with an isocaloric diet restricted in carbohydrates, suggesting a LF diet to be more effective for weight loss compared with a low carbohydrate diet. Our results from mice also demonstrate a greater fat mass loss during the intake of LF diets compared with HF/HP diets. Furthermore, our results from calorie restricted HF/HP-fed mice are in agreement with the recent finding that a HF intake (64E%) for 6 weeks does not impair insulin sensitivity in healthy, slightly overweight men in caloric balance [49].

The protein sources used in this study were restricted to different animal-derived proteins, casein, cod and pork, and thus we do not examine plant or insect proteins—protein sources of interest in relation to more sustainable development. However, plant-based protein sources would also be of interest to evaluate, as studies have demonstrated that the metabolic impact from plant-based proteins differ from animal derived proteins [28,50]. Increasing the length of the experimental study could potentially reveal additional effects between the different animal proteins used, especially the 6 week intervention in the obese mice which may have been too short to induce significant metabolic alterations. 

## 5. Conclusions

In already obese animals, the impact of the protein source on obesity varies depending on energy intake and macronutrient composition, whereas glucose tolerance and insulin sensitivity in general follow the differences detected in obesity development. Combined, our results demonstrate that different animal protein sources may have a greater impact during obesity development than during obesity reversal. Macronutrient composition and energy access modulate the impact of dietary animal protein sources in obese mice. The dietary protein source significantly impacts the order of magnitude for changes in body weight, fat mass, lean mass, feed efficiency, glucose tolerance and insulin sensitivity in already obese animals fed HF/HP diets *ad libitum* or LF diets with or without energy restriction. All the dietary interventions combined with calorie restriction from our study led to a considerable body weight loss of obese mice, as in agreement with a number of human weight loss interventions [46,51,52,53,54,55,56]. However, our study also demonstrated that the protein source appears to be of no importance when combined with energy restriction and HF/HP intake, hence, the results indicate that the dietary protein source is of greater importance in preventing weight gain than reversing obesity. It may be considered whether dietary means for obesity prevention in lean persons should be different from dietary advice given to obese subjects to achieve effective weight loss. On the basis of the high number of adults and children currently being overweight or obese, the need for effective dietary strategies to prevent, stabilize and reduce weight is urgent. Detecting differences in efficiency between dietary strategies and dietary sources for weight gain prevention and weight loss are of importance for dietary recommendations.

## Figures and Tables

**Table 1:** Dietary composition of the HF/HS ^1^ diet and the HF/HP ^2^ diets.

Component (g/kg)	HF/HS	HF/HP Casein	HF/HP Cod	HF/HP Pork	LF ^3^ Casein	LF Cod	LF Pork
Casein ^#^	69	416			207		
Cod ^#^	67		431			215	
Pork ^#^	73			461			217
Potato starch (dextrin)	11	21	9,31	0	531	524	530
Sucrose	439	214	214	214	92	92	92
Corn oil	242	248	245	224	69	69	61
Cellulose	50	50	50	50	50	50	50
*t*-Butylhydroquinone	0.014	0.014	0.014	0.014	0.014	0.014	0.014
Mineral mix: SDS, AIN93Gminmix	35	35	35	35	35	35	35
Vitamin mix: SDS, AIN93VX NCR95compliant	10	10	10	10	10	10	10
Choline bitartrate	2.5	2.5	2.5	2.5	2.5	2.5	2.5
L-Cystine	3	3	3	3	3	3	3
Total	1000	1000	1000	1000	1000	1000	1000

^1^ High-fat/high-sucrose (HF/HS), ^2^ high-fat/high-protein (HF/HP), ^3^ low-fat (LF) diets ^#^ added to the diets in amounts equal to 200 g crude protein/kg (33 E%) for the LF diets and 400 g crude protein/kg (16 E%) for the HF/HP diet. SDS—Special Diets Services.

## Supplementary Materials

The following are available online at https://www.mdpi.com/2072-6643/11/5/1153/s1, Figure S1: (**a**) Hematoxylin and eosin (H&E) staining of inguinal white adipose tissue (iWAT) after 12 weeks of feeding *ad libitum* high-fat/high-protein (HF/HP) diets based on different protein sources to lean mice (scalebar = 100μm). (**b**) Mean cell diameter ± SEM of adipocytes from iWAT (*n* = 4). (**c**) Expression levels of the brown adipose tissue marker genes uncoupling protein 1 (*Ucp1*), deiodinase iodothyronine type II (*Dio2*) and peroxisome proliferator-activated receptor-g coactivator 1a (*Ppargc1a*) in iWAT (*n* = 5–7). Expression levels are normalized to the gene expression of TATA-box binding protein (*Tbp*). (**d**) H&E staining of interscapular brown adipose tissue (iBAT) after 12 weeks of feeding *ad libitum* HF/HP diets based on different protein sources to lean mice (scalebar = 100μm). (**e**) Immunohistochemical staining with UCP1-antibody and (**f**) quantification of percent area stained with UCP1-antibody demonstrated as mean ± SEM (*n* = 3–5). Different letters denote significant differences (*P* < 0.05) by one-way ANOVA using uncorrected Fisher’s LSD multiple comparison. Figure S2: (**a**) Total energy intake (kcal) after 12 weeks, (**b**) apparent fat digestibility (%) and (**c**) apparent nitrogen digestibility (%) after 6 weeks of feeding ad libitum high-fat/high-protein (HF/HP) diets based on different protein sources to lean mice. Data are presented as mean ± SEM (*n* = 8–9) and different letters denote significant differences (*P* < 0.05) by one way ANOVA using uncorrected Fisher’s LSD multiple comparison. Figure S3: (**a**) Hematoxylin and eosin (H&E) staining of interscapular brown adipose tissue (iBAT) after 6 weeks of feeding high-fat/high-protein (HF/HP) diets based on different protein sources *ad libitum* to already obese mice, in addition to one group fed the obesogenic high fat high sucrose (HF/HS) diet for 6 weeks (scalebar = 100μm). (**b**) Immunohistochemical staining with UCP1-antibody and (**c**) quantification of percent area stained with UCP1-antibody demonstrated as mean ± SEM (*n* = 3–4). (**d**) HE staining of inguinal white adipose tissue (iWAT) (scalebar = 100μm). (**e**) Mean cell diameter (± SEM) of adipocytes from iWAT (*n* = 4–5). Figure S4: Expression levels of brown adipose tissue marker genes (uncoupling protein 1 (*Ucp1*), deiodinase iodothyronine type II (*Dio2*), peroxisome proliferator-activated receptor-g coactivator 1a (*Ppargc1a*)) and *leptin* in (**a**) interscapular brown adipose tissue (iBAT) and (**b**) inguinal white adipose tissue (iWAT) after 6 weeks of feeding highfat/high-protein (HF/HP) diets based on different protein sources *ad libitum* or with 30 percent calorie restriction (CR) to already obese mice, in addition to one group fed the obesogenic high-fat/high-sucrose (HF/HS) diet (*n* = 7–13). Expression levels are normalized to gene expression of TATA-box binding protein (*Tbp*). Figure S5: Hematoxylin and eosin (H&E) staining of interscapular brown adipose tissue (iBAT) after 6 weeks of feeding high-fat/high-protein (HF/HP) diets based on different protein sources with 30 per cent calorie restriction (CR) to already obese mice, in addition to one group fed the obesogenic high-fat/highsucrose (HF/HS) diet (scalebar = 100μm). Figure S6: Expression levels of brown adipose tissue marker genes (uncoupling protein 1 (*Ucp1*), deiodinase iodothyronine type II (*Dio2*), peroxisome proliferator-activated receptor-g coactivator 1a (*Ppargc1a*)), in addition to *leptin* and glycerol kinase (*Gk*) in (**a**) inguinal white adipose tissue (iWAT) and (**b**) interscapular brown adipose tissue (iBAT) after 6 weeks of feeding low-fat (LF) diets based on different protein sources *ad libitum* or with 30 percent calorie restriction (CR) to already obese mice, in addition to one group fed the obesogenic very high-fat (VHF) diet *ad libitum* (*n* = 8–10). Expression levels are normalized to gene expression of TATA-box binding protein (*Tbp*). Table S1: Primer sequences used for quantitative reverse transcriptase PCR.
