# Supplementary material for: The Impact of Different Animal-Derived Protein Sources on Adiposity and Glucose Homeostasis during Ad Libitum Feeding and Energy Restriction in Already Obese Mice
Source: Nutrients. 2019 May 23;11(5):1153. doi: 10.3390/nu11051153 (PMC6567247; doi:10.3390/nu11051153)
Supplement: Supplementary file 1 [file nutrients-11-01153-s001.zip › Figure S1.pdf]

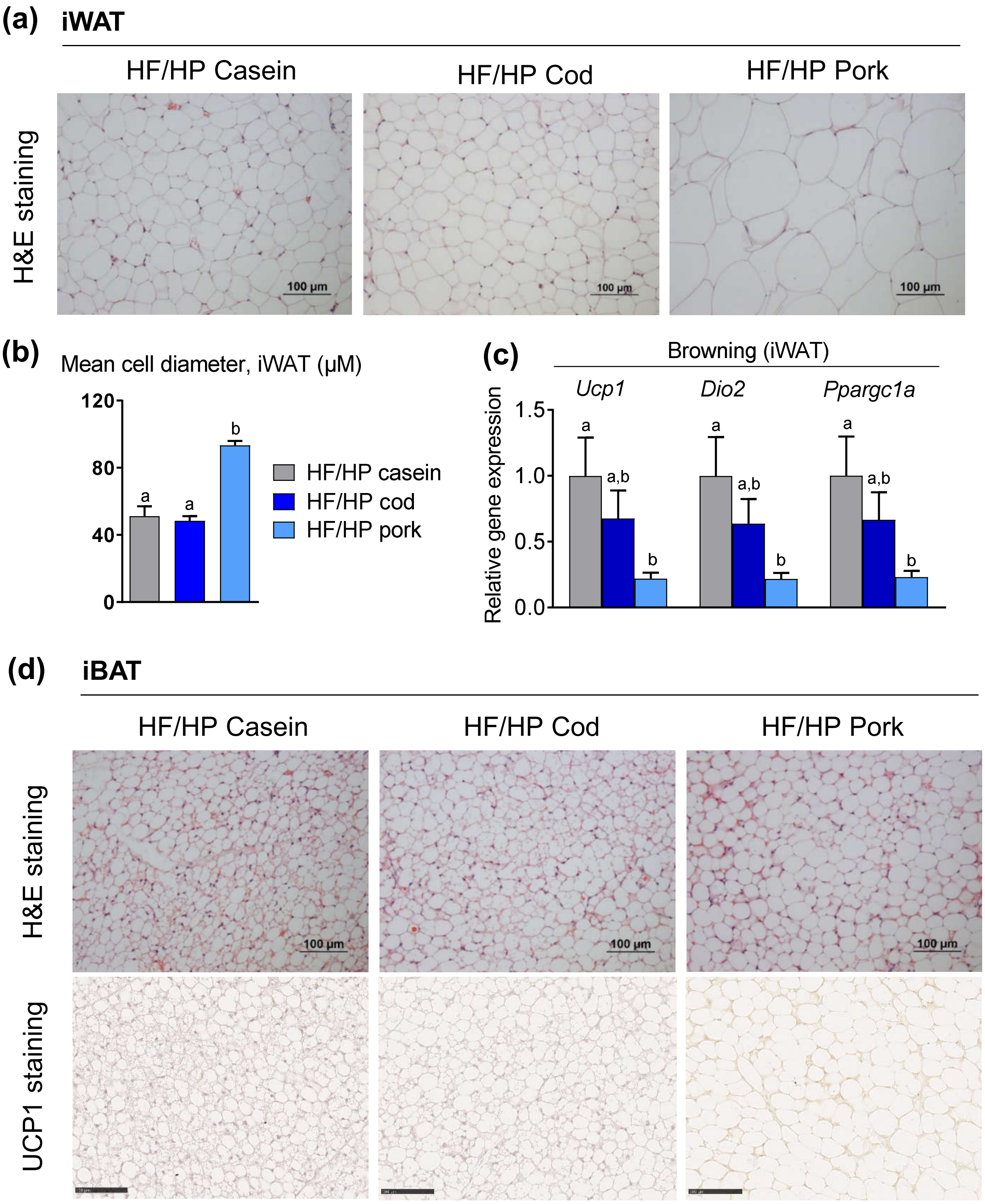

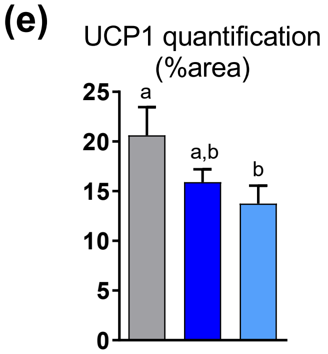

Figure S1. **(a)** Hematoxylin and eosin (H&E) staining of inguinal white adipose tissue (iWAT) after 12 weeks of feeding *ad libitum* high-fat/high-protein (HF/HP) diets based on different protein sources to lean mice (scalebar = 100μm). **(b)** Mean cell diameter ± SEM of adipocytes from iWAT (n=4). **(c)** Expression levels of the brown adipose tissue marker genes Uncoupling protein 1 *(Ucp1)*, Deiodinase iodothyronine type II *(Dio2)*, Peroxisome proliferator-activated receptor-g coactivator 1a *(Ppargc1a)* in iWAT (n=5-7). Expression levels are normalized to the gene expression of TATA-box binding protein *(TBP)*. **(d)** H&E staining of interscapular brown adipose tissue (iBAT) after 12 weeks of feeding *ad libitum* HF/HP diets based on different protein sources to lean mice (scalebar = 100μm). **(e)** Immunohistochemical staining with UCP1-antibody and **(f)** quantification of per cent area stained with UCP1-antibody demonstrated as mean ± SEM (n=3-5). Different letters denote significant differences ($P<0.05$) by one-way ANOVA using uncorrected Fisher's LSD multiple comparison.
