# Supplementary material for: The Impact of Different Animal-Derived Protein Sources on Adiposity and Glucose Homeostasis during Ad Libitum Feeding and Energy Restriction in Already Obese Mice
Source: Nutrients. 2019 May 23;11(5):1153. doi: 10.3390/nu11051153 (PMC6567247; doi:10.3390/nu11051153)
Supplement: Supplementary file 1 [file nutrients-11-01153-s001.zip › Figure S2.pdf]

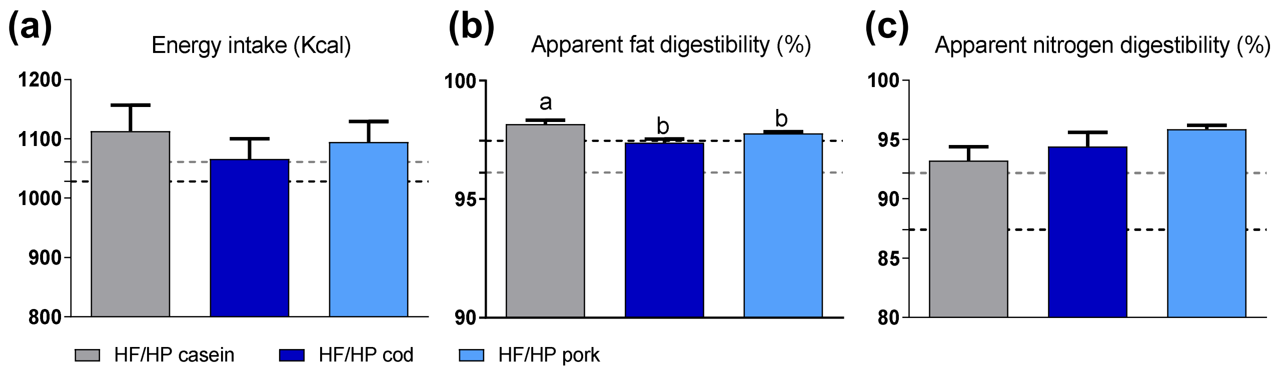

Figure S2. **(a)** Total energy intake (kcal) after 12 weeks, **(b)** apparent fat digestibility (%) and **(c)** apparent nitrogen digestibility (%) after 6 weeks of feeding *ad libitum* high-fat/high-protein (HF/HP) diets based on different protein sources to lean mice. Data are presented as mean ± SEM (n=8-9) and different letters denote significant differences (P<0.05) by one-way ANOVA using uncorrected Fisher's LSD multiple comparison.
