# Supplementary material for: The Impact of Different Animal-Derived Protein Sources on Adiposity and Glucose Homeostasis during Ad Libitum Feeding and Energy Restriction in Already Obese Mice
Source: Nutrients. 2019 May 23;11(5):1153. doi: 10.3390/nu11051153 (PMC6567247; doi:10.3390/nu11051153)
Supplement: Supplementary file 1 [file nutrients-11-01153-s001.zip › Figure S3.pdf]

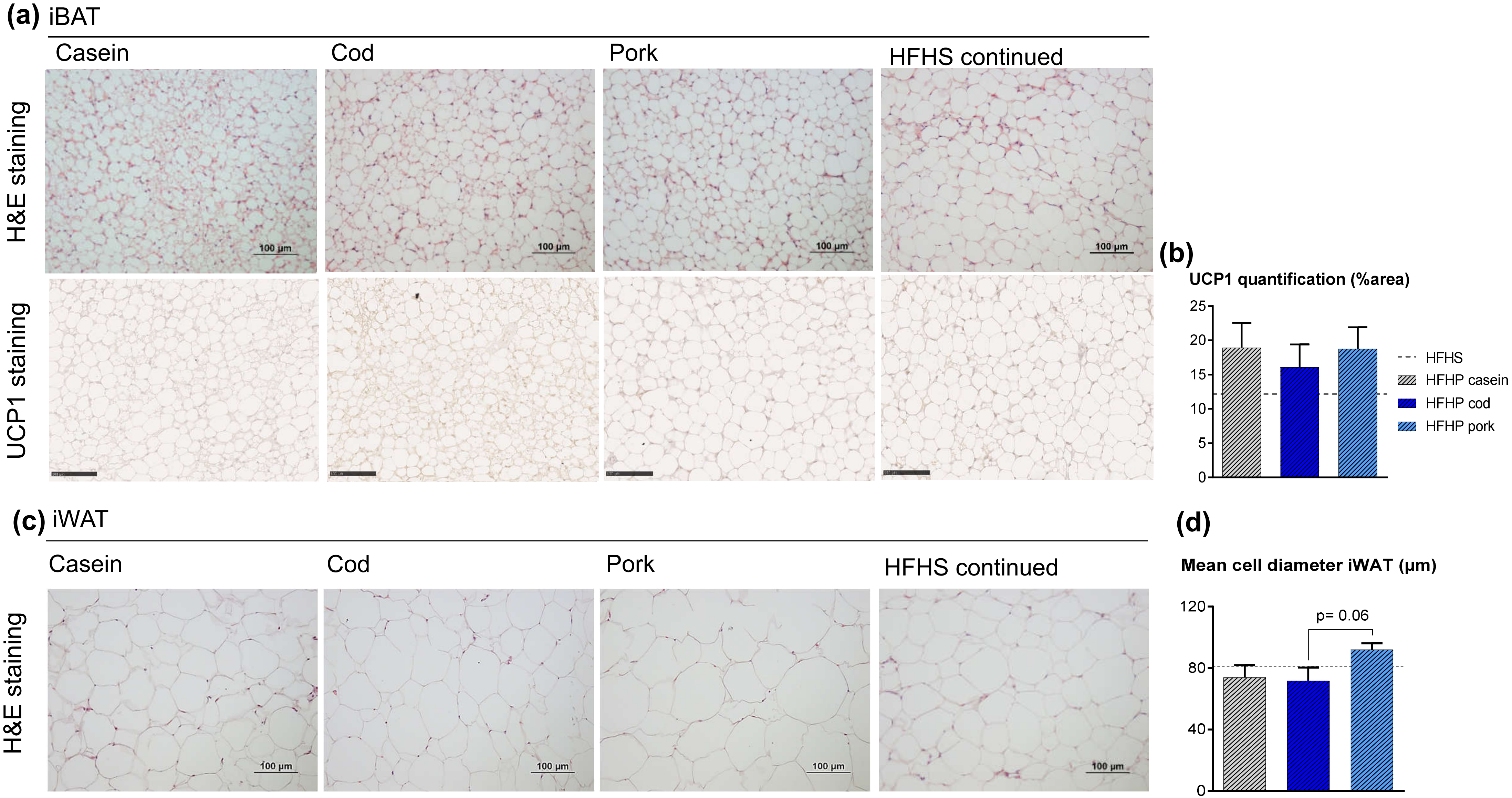

Figure S3. (a) Hematoxylin and eosin (H&E) staining of interscapular brown adipose tissue (iBAT) after 6 weeks of feeding high-fat/high-protein (HF/HP) diets based on different protein sources *ad libitum* to already obese mice, in addition to one group fed the obesogenic high fat high sucrose (HF/HS) diet for 6 weeks (scalebar = 100µm). **(b)** Immunohistochemical staining with UCP1-antibody and **(c)** quantification of per cent area stained with UCP1-antibody demonstrated as mean ± SEM (n=3-4). **(d)** HE staining of inguinal white adipose tissue (iWAT) (scalebar = 100µm). **(e)** Mean cell diameter (± SEM) of adipocytes from iWAT (n=4-5).
