# Supplementary material for: The Impact of Different Animal-Derived Protein Sources on Adiposity and Glucose Homeostasis during Ad Libitum Feeding and Energy Restriction in Already Obese Mice
Source: Nutrients. 2019 May 23;11(5):1153. doi: 10.3390/nu11051153 (PMC6567247; doi:10.3390/nu11051153)
Supplement: Supplementary file 1 [file nutrients-11-01153-s001.zip › Figure S5.pdf]

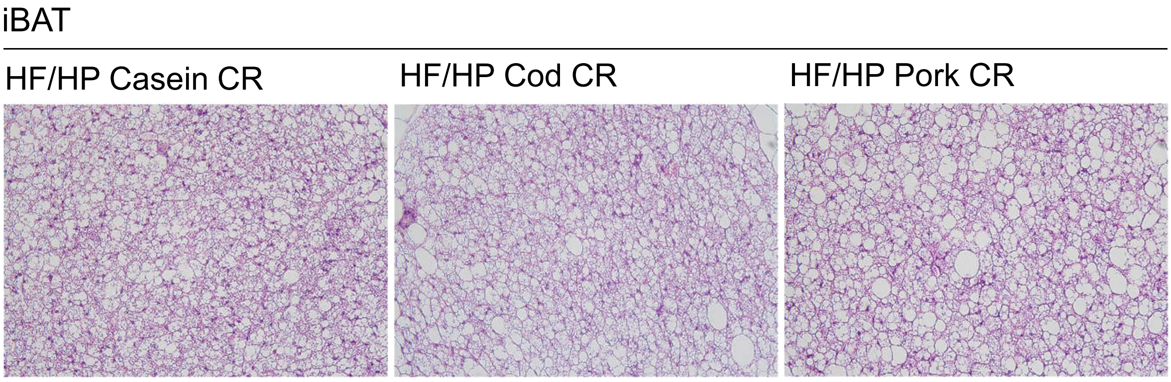

Figure S5. Hematoxylin and eosin (H&E) staining of interscapular brown adipose tissue (iBAT) after 6 weeks of feeding high-fat/high-protein (HF/HP) diets based on different protein sources with 30 per cent calorie restriction (CR) to already obese mice, in addition to one group fed the obesogenic high-fat/high-sucrose (HF/HS) diet (scalebar = 100μm).
