# Supplementary material for: The Impact of Different Animal-Derived Protein Sources on Adiposity and Glucose Homeostasis during Ad Libitum Feeding and Energy Restriction in Already Obese Mice
Source: Nutrients. 2019 May 23;11(5):1153. doi: 10.3390/nu11051153 (PMC6567247; doi:10.3390/nu11051153)
Supplement: Supplementary file 1 [file nutrients-11-01153-s001.zip › Figure S6.pdf]

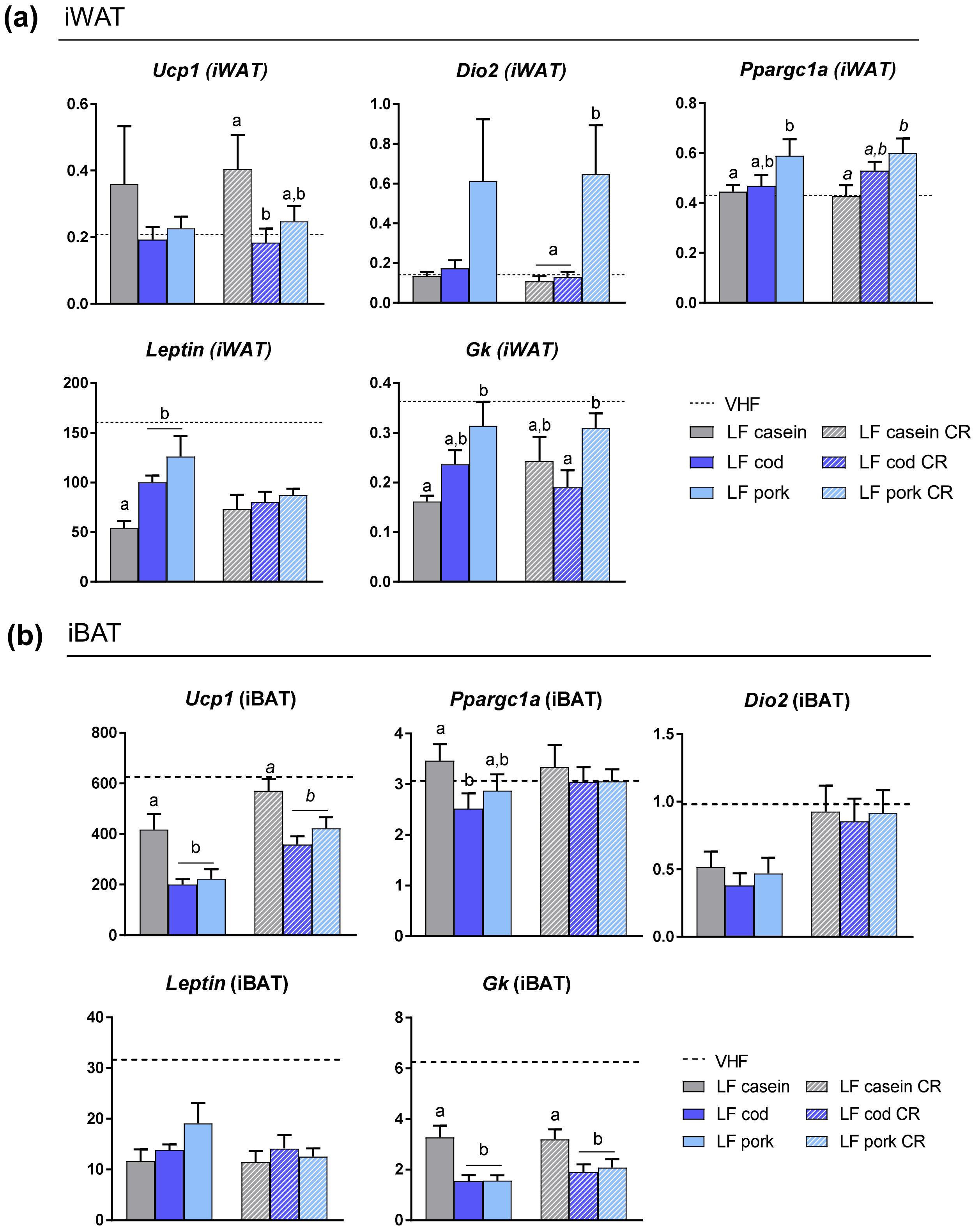

Figure S6. Expression levels of brown adipose tissue marker genes (uncoupling protein 1 *(Ucp1)*, deiodinase iodothyronine type II *(Dio2)*, peroxisome proliferator-activated receptor-g coactivator 1a *(Ppargc1a)*), in addition to *Leptin* and Glycerol kinase (*Gk*) in **(a)** inguinal white adipose tissue (iWAT) and **(b)** interscapular brown adipose tissue (iBAT) after 6 weeks of feeding low-fat (LF) diets based on different protein sources *ad libitum* or with 30 per cent calorie restriction (CR) to already obese mice, in addition to one group fed the obesogenic very high-fat (VHF) diet *ad libitum* (n=8-10). Expression levels are normalized to gene expression *of* TATA-box binding protein *(TBP)*.
