# Supplementary material for: The Impact of Different Animal-Derived Protein Sources on Adiposity and Glucose Homeostasis during Ad Libitum Feeding and Energy Restriction in Already Obese Mice
Source: Nutrients. 2019 May 23;11(5):1153. doi: 10.3390/nu11051153 (PMC6567247; doi:10.3390/nu11051153)
Supplement: Supplementary file 1 [file nutrients-11-01153-s001.zip › Table S1.pdf]

Table S1. Primer sequences used for quantitative reverse transcriptase PCR.

Gene name (*abbreviation*)	Accession number	Forward primer sequence	Reverse primer sequence
Uncoupling protein 1 *(Ucp1)*	NM_009463.3	AGCCGGCTTAATGACTGGAG	TCTGTAGGCTGCCCAATGAAC
Deiodinase iodothyronine type II *(Dio2)*	NM_010050.2	GCCCAGCAAATGTAGAC	TGGCAATAAGGAGCTAGAA
Peroxisome proliferator-activated receptor-g coactivator 1a *(Ppargc1a)*	NM_008904.2	CATTTGATGCACTGACAGATGGA	CCGTCAGGCATGGAGGAA
Leptin	NM_008493.3	ATTTCACACACGCAGTCGGTAT	AAGCCCAGGAATGAAGTCCA
Glycerol kinase (*Gk*)	NM_001294138.1	CAAATGCAAGCAGGACGATG	GGCCCCAGCTTTCATTAGG
TATA-Box Binding Protein *(TBP)*	NM_013684.3	ACCCTTCACCAATGACTCCTATG	ATGATGACTGCAGCAAATCGC
